# Abnormalities in Alternative Splicing of Apoptotic Genes and Cardiovascular Diseases

**DOI:** 10.3390/ijms161126017

**Published:** 2015-11-13

**Authors:** Zodwa Dlamini, Shonisani C. Tshidino, Rodney Hull

**Affiliations:** 1 Research, Innovation and Engagements, Mangosuthu University of Technology, Durban 4026, South Africa; 2 Department of Biochemistry, Microbiology and Biotechnology, University of Limpopo, Polokwane 0727, South Africa; shonisani.tshidino@ul.ac.za; 3 College of Agriculture and Environmental Sciences, Department of Life and Consumer Sciences, Florida Science Campus, University of South Africa, Johannesburg 1709, South Africa; hullr@unisa.ac.za

**Keywords:** apoptosis, alternative splicing, cardiomyopathies, *PUMA*, *Bcl-2*, *Bnip3*, *Nix*, therapeutic strategies for targeting heart disease associated abnormal splicing

## Abstract

Apoptosis is required for normal heart development in the embryo, but has also been shown to be an important factor in the occurrence of heart disease. Alternative splicing of apoptotic genes is currently emerging as a diagnostic and therapeutic target for heart disease. This review addresses the involvement of abnormalities in alternative splicing of apoptotic genes in cardiac disorders including cardiomyopathy, myocardial ischemia and heart failure. Many pro-apoptotic members of the *Bcl-2* family have alternatively spliced isoforms that lack important active domains. These isoforms can play a negative regulatory role by binding to and inhibiting the pro-apoptotic forms. Alternative splicing is observed to be increased in various cardiovascular diseases with the level of alternate transcripts increasing elevated in diseased hearts compared to healthy subjects. In many cases these isoforms appear to be the underlying cause of the disease, while in others they may be induced in response to cardiovascular pathologies. Regardless of this, the detection of alternate splicing events in the heart can serve as useful diagnostic or prognostic tools, while those splicing events that seem to play a causative role in cardiovascular disease make attractive future drug targets.

## 1. Introduction

**Figure 1:**
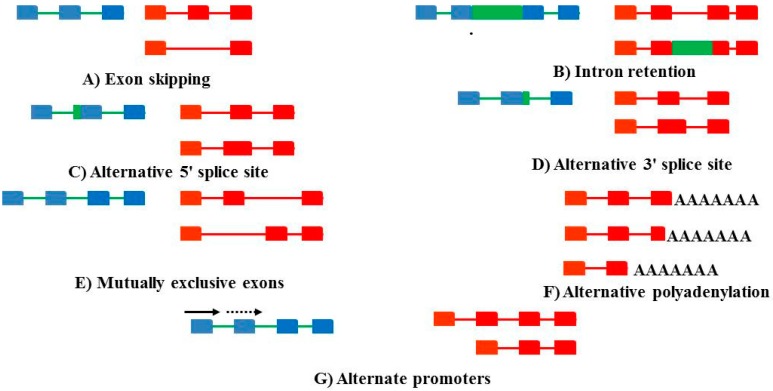
Various forms of alternate splicing. The genomic sequences are marked in blue with the exons appearing as blue blocks and the introns marked in green. The transcripts are in red. (**A**) Exon skipping occurs when an exon is excluded (**B**) Intron retention occurs when an intron is retained and not excised (**C**,**D**). Alternative splice sites involve the 5′ and 3′ exons being shortened due to internal splice sites. (**E**) Mutually exclusive exons imply that one exon is included, whereas another is excised. The inclusiom of exon 1 (the solid arrow) precludes the inclusion of the second exon (dashed arrow) (**F**) Alternative polyadenylation sites can change the length of the 3′ untranslated region. And (**G**) alternate promoters can change transcription initiation sites.

Alternative splicing is a posttranscriptional action that occurs in eukaryotic organisms, wherein a single gene produces multiple distinct transcripts [1]. In this manner, a single pre-mRNA transcript can be alternatively spliced to produce multiple mature mRNA splice variants, which are then translated into varying protein isoforms. Alternative splicing is controlled by competition among the spliceosomes, splice sites and splicing elements for splicing factors (Reviewed in [2]). There are several means whereby this can be achieved (Figure 1). Alternative splicing could also down-regulate gene expression via the introduction of premature stop codons, which result in nonsense-mediated decay of the transcript (mRNA). Protein produced by the resulting mRNA isoforms may differ in activity or function, structure, localization or in other ways [2]. It is now well known that up to 92%–94% human multi-exon genes undergo alternative splicing [3]. Since RNA binding proteins, including putative splicing factors are differentially expressed and spliced across tissues at deferent developmental stages, such proteins may be involved in regulating tissue and temporal variation in isoform expression. In addition, it has also been suggested that alternative splicing is a critical developmental regulatory mechanism [4]. Alternative splicing may be controlled by *cis* and *trans* elements. *Trans* elements, mostly RNA-binding proteins regulate the activity of spliceosome and *cis* elements; Serine/arginine-rich (SR) proteins and the heterogeneous nuclear ribo-nucleoproteins (hnRNP) family of proteins (Reviewed in [1,5]). The regulation of alternative splicing is tightly governed, with errors in splicing regulation resulting in disease occurrence (Reviewed in [6,7]).

Alternative splicing is known to play an important regulatory role in apoptosis, with many apoptosis genes being alternatively spliced, and some isoforms often playing an antagonistic role [8]. Multiple splice variants exist for members of the *Bcl-2* family genes such as *Bcl-x* and *PUMA* as well as for caspases such as caspase-2 and caspase-3 [9,10,11].

Apoptosis is defined as the removal of individual cells after their fragmentation into membrane-bound particles, which are then phagocytosed by specialized cells such as macrophages and neutrophils [9]. Apoptosis facilitates the removal of infected, damaged, aged, or dangerous cells in order to limit disruption of surrounding tissues and for maintenance of cells normal development and balance tissue homeostasis [12,13]. Hence, a failure to correctly execute apoptosis and the subsequent clearance of cells that have undergone apoptosis is generally associated with autoimmune and/or chronic inflammatory diseases [14].

**Figure 2:**
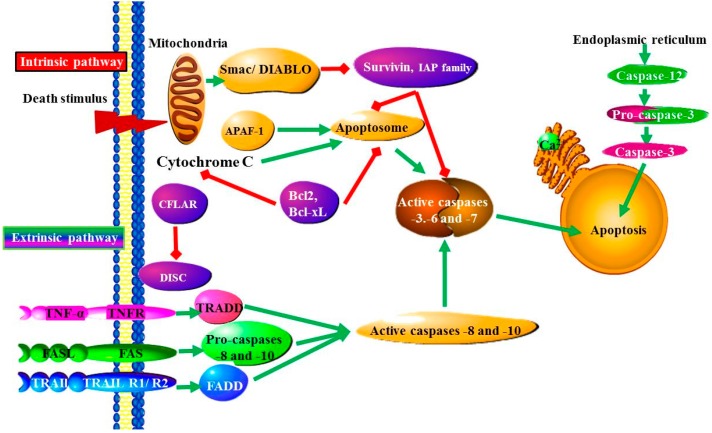
The Intrinsic and extrinsic apoptotic pathways: The intrinsic, or mitochondrial, pathway is initiated from within the cell in response to DNA damage, loss of cell-survival factors and severe cell stress. Damaged mitochondria release proteins, such as SMAC (second mitochondria-derived activator of caspase)/Diablo, which counteract the effect of IAPs (inhibitor of apoptosis proteins), which normally bind and prevent activation of caspase-3. The extrinsic pathway begins outside the cell through activation of pro-apoptotic receptors on the cell surface. Ligand binding causes Death receptors to cluster and form a death-inducing signaling complex (DISC). This complex activates caspase-8, which activates downstream caspases-3 and -7, committing the cell to apoptosis. Cellular stress induced apoptosis occurs by a mechanism that involves altering mitochondrial permeability and subsequent cytochrome c release and formation of the apoptosome, which activates caspase-9. Activated caspase-9 then cleaves caspase-3 resulting in downstream events involved in cell death. Release of cytochrome c is regulated by Bcl-2 family proteins. The released cytochrome c binds to apoptotic protease activating factor-1 (APAF1) and activates caspase-9 [21].

The apoptotic process is mainly characterized by structural changes, DNA fragmentation, cytoplasmic and nuclear condensation, chromatin condensation, phosphatidylserine extrusion, vacuolization and the formation of apoptotic bodies. In eukaryotes, apoptosis is critical for an effective immune system, normal development, maintenance of tissue homeostasis, embryonic development and chemical (drug)-mediated cell death [14,15]. Two pathways exist, whereby a cell can undergo apoptosis, namely the (i) extrinsic and (ii) intrinsic pathways (Figure 2). The pathway that the cell actually uses to undergo apoptosis is dependent on the pathological condition and tissue type. Briefly, intrinsic pathways are induced by either stress to the Endoplasmic reticulum (ER) or DNA damage [16]. Amongst other responses, DNA damage stimulates the release of p53 that can result in mitochondrial membrane dysfunction, whereas ER stress mediates calcium accumulation and calpain activation, which can result in lysosomal rupture, cathepsin release or the activation of caspases [16]. Extrinsic pathways involve death-receptor activation and the withdrawal of survival factors. Death-receptors are mainly activated by certain membrane receptors such as Fas and TNF-α. The latter involves activation of JNK and c-Jun by inflammatory cytokines, Reactive oxygen species (ROS), mixed lineage kinases, radiation or excitotoxicity [16]. In both pathways, cytochrome c is released with activation of down-stream caspases and cell death. However, the release of apoptosis-inducing factor (AIF) or endonuclease G (Endo G) factors from mitochondria does not involve caspase activation during induction of cellular damage and apoptosis. Thus, both pathways subsequently activate certain cascades of factors, which eventually result in cell death via their effects on mitochondrial membrane stability, *i.e.*, increase in pro-apoptotic genes, and decrease in anti-apoptotic genes as well as activation of caspases [5,8,16].

Apoptotic pathways play a major role in the development of cardiovascular disease. The loss of terminally differentiated adult cardiomyocytes is a major contributor to heart disease, while controlled apoptosis is necessary for heart development where it is involved in forming the right ventricle. Additionally, in response to the loss of cardiomyocytes, the body deposits collagen, which leads to increased cardiomyocyte wall stress, and impaired ventricular relaxation [17]. Hence, abnormalities in regulation of alternative splicing of apoptotic genes remain a major route in the pathogenesis of several diseases including dilated cardiomyophathy, diabetic cardiomyopathy, atherosclerosis, myocardial ischemia/reperfusion injury and chronic heart failure (Reviewed in [18,19,20]). It has been long recognized that elucidation of apoptosis pathways as well as their regulators can offer novel therapeutic targets and prognosis of various heart diseases [17]. Thereafter, both extrinsic and intrinsic pathways of apoptosis have been found to be activated in heart failure as a result of heart disease [18].

## 2. Cardiomyopathies that Have Apoptosis Related Causes

**Figure 3:**
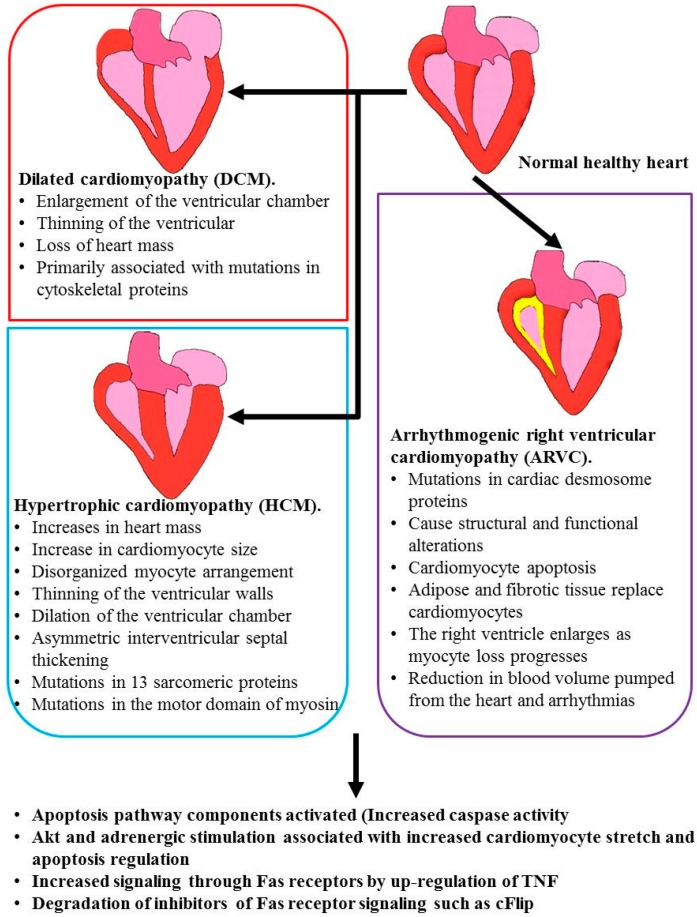
Common cardiac pathologies associated with aberrant apoptosis: Loss of regulation of apoptotic pathways is an underlying cause of many heart pathologies.

Presently, heart failure is the leading cause of morbidity and mortality worldwide [22]. The heart is composed of cardiomyocytes, fibroblasts, endothelial and smooth muscle cells, while the central unit of contraction in the cardiomyocytes, the sarcomere, is made of interdigitating thick and thin filaments, which generate force by cyclical cross-bridge formation between actin and myosin [23]. Mutations in the genes coding for both the proteins that make up the sarcomere as well as those involved in cardiac regulation contribute to the susceptibility of the heart to malfunctions [24]. Heart failure can be caused by a variety of cardiovascular diseases such as hypertensive heart failure, dilated cardiomyopathy, rheumatic heart disease, congenital heart disease, myocardial ischemia/reperfusion injury and coronary artery (Figure 3).

### 2.1. Ischemia/Reperfusion Injury

Myocardial ischemia occurs when there is an inadequate supply of oxygen and glucose to the heart. This may lead to cardiomyocyte death, however the rapid restoration of oxygen and glucose (reperfusion) is also associated with cell death [17]. Studies implicate that both apoptosis and necrosis play a role in ischemia/reperfusion cell death [21,25]. Early studies defined a separate role for necrosis and apoptosis, with necrosis being the prevalent form of cell death in ischemia, while apoptosis was the dominant form during reperfusion injury. This appears to be due to apoptosis being an ATP (energy) dependent process and therefore, requiring a supply of both oxygen and glucose [21]. However, other studies refuted this finding and concluded that apoptosis was responsible for the initial cell death, which was then maintained through necrosis [25] or that apoptosis and necrosis occurred simultaneously [18,22]. Apoptosis in response to Ischemia involves both the intrinsic and extrinsic apoptotic pathways, with null *Fas* mutant mice being partially protected from ischemia and reperfusion induced cell death [26], while deletion of either *TNFR1* or *TNFR2* had no effect on the extent of the apoptotic response [27]. The extent of ischemia or reperfusion induced apoptosis was also reduced through the overexpression of Bcl-2, IAP-2 and apoptosis repressor with caspase recruitment domain (ARC). Similar results were achieved through the deletion of *Bax* and *PUMA* (Reviewed in [22,28]).

### 2.2. Hypertrophic Cardiomyopathy (HCM) and Dilated Cardiomyopathy (DCM)

The activation of apoptotic pathways and increased caspase activity has been observed in both hypertrophic cardiomyopathy (HCM) and dilated cardiomyopathy (DCM) [29,30,31] (Figure 3). HCM is inherited in an autosomal dominant pattern in more than 50% of patients and is most commonly associated with mutations in 13 sarcomeric proteins. Most of these mutations occur in the motor domain of myosin, the first mutations were identified in the gene encoding myosin heavy chain β (MYH7), but also occur in the tail region [23]. Mutations in the cardiac myosin binding protein c gene are also common [32]. HCM results in the initial increase in heart mass and inter-ventricle septal thickening. An obstruction in the outflow of blood is also common, being present in 25% of cases. Diastolic dysfunction arises due to left ventricular stiffness. HCM is also characterized by an increase in the size of cardiomyocytes, myofibrillar disarray, thinning ventricular walls and dilation of the ventricular chamber [23,32].

Dilated cardiomyopathy (DCM) is associated with the loss of heart mass due to ventricular chamber enlargement and thinning of the ventricular walls. DCM is an autosomal mutation transmitted through maternal mitochondrial DNA, or as an X-linked mutation and these mutations are primarily in genes encoding cytoskeletal proteins [23]. Mutations in N-cadherin that result in a loss of function may lead to an increase in apoptosis as N-cadherin-mediated adhesion protects melanoma cells from apoptosis by activating the anti-apoptotic Akt (PKB) pathway [19]. Chronically activated Akt causes cardiomyopathy, with constitutively activated Akt in mice resulting in hearts and cardiomyocytes two-fold larger than littermate controls [33]. Akt is expressed as three different isoforms, Akt1, Akt2 and Akt3. These Akt isoforms play unique roles in the regulation of apoptosis in different tissue types and genetic backgrounds [34]. For instance, the X-linked inhibitor of apoptosis protein (XIAP) is regulated by both Akt1 and Akt2. Additionally, Akt2 knockdown in cancer cells resulted in decreased proliferation, while Akt1 knockdown in the same tissue, had no observable effect [34].

### 2.3. Arrhythmogenic Right Ventricular Cardiomyopathy (ARVC)

Arrhythmogenic right ventricular cardiomyopathy (ARVC) is inherited in an autosomal dominant or recessive pattern and is caused by mutations in the genes coding for proteins that make up the cardiac desmosome [23] (Figure 3). These mutated proteins lead to a disruption in desmosome structure and function, which leads to cardiomyocyte apoptosis [35]. The lost cardiomyocytes are replaced by adipose and fibrotic tissue, and this occurs primarily in the right ventricle. The loss of myocytes from the right ventricle leads to ventricle enlargement, causing a reduction in blood volume pumped from the heart and arrhythmias [23].

### 2.4. Hypoplastic Left Heart Syndrome (HLHS)

Hypoplastic left heart syndrome (HLHS) is characterized by right ventricle failure due to pressure and volume overload, as well as hypoxia. This is due to underdevelopment of the left ventricle [36]. Over 180 changes in alternate splicing events were detected in tissue from the right ventricle of patients with HLHS. These included changes in the splicing of transcripts involved in cell metabolism, the spliceosome, structural proteins and calcium transport channels [36]. The underdevelopment of the left ventricle is due to increased levels of apoptosis that occur during the development of the left ventricle, changing the proliferation level of the cardiomyocytes [37,38].

## 3. The Impact of Alternative Splicing on Cardiac Disease

Alternative splicing is a known underlying cause for many cardiac diseases. Myotonic dystrophy type1 (DM1) is a common Inherited neuromuscular disease caused by a mutation in the gene encoding dystrophia myotonica protein kinase (DMPK). This results in the accumulation of mutant mRNA within the nucleus, which leads to aberrant alternative splicing and multiple pathologies including heart disease. One heart disease where alternative splicing is implicated as the underlying cause is Brugada syndrome. This is a life-threatening heart rhythm disorder that is the result of the abnormal splicing of *SCN5A*, the gene encoding the Na^+^ channel subunit expressed in the human heart [39].

Abnormalities in alternative splicing of four sarcomere genes in dilated cardiomyopathy and ischemic cardiomyopathy have been reported and that their abnormalities precede the onset of heart failure [40]. The accuracy with which samples could be divided as originating from healthy or unhealthy patients, based on the occurrence of splice variants was high enough that these splicing alterations could be used as diagnostic tools [40]. Apoptotic pathways and mutations in sarcomeric proteins are both observed in hypertrophic cardiomyopathy (HCM) [23,29,30,31]. Mutations in the anti-apoptotic gene *BAG3* result in increased apoptosis and impaired Z disc assembly [41]. Drugs that induce apoptosis in cardiomyocytes also lead to a reduction in the expression of sarcomeric proteins [42].

Both myosin heavy chain 7 (MYHC7) and troponin I, cardiac muscle (TNNI3) showed an increase in the level of alternatively spliced transcripts in cases of heart disease. These were intron retention events [40]. Overuse of cryptic splice sites within the *MHH7* gene lead to the formation of a transcript lacking eight exons. This splicing event is thought to occur due to the altered DNA methyltransferase function in diseased hearts [43]. The importance of the alternative splicing of sarcomere gene transcripts in cardiomyopathies is further demonstrated by mutations in the gene encoding the RNA-binding protein RBM20 causing dilated cardiomyopathy (DCM). The mutant form of this protein alters cardiac RNA splicing. Some of the targets of RBM20 include the sarcomere proteins Titin, myosin heavy chain 7, myomesin, nexillin, obscurin and troponin as well as the cytoskeletal proteins dystonin [44].

Atherosclerosis, the accumulation of degenerative material in the inner layer of artery cell walls, is a leading cause of heart disease with plaque rupture caused by angiogenesis being the cause of many of these coronary events. The tissue factor gene plays a role in angiogenesis and a splice isoform of this protein known as alternatively spliced tissue factor (asTF) is expressed in these vulnerable plaques resulting in increased angiogenesis due to increased vascular endothelial growth factor expression through up-regulated hypoxia-inducible factor-1α [45].

### 3.1. Hypertrophic Cardiomyopathy

The influence of alternative splicing on hypertrophic cardiomyopathy is seen in the alternative splicing of the sarcomere proteins myomesin [46] and troponin [47]. Dilated cardiomyopathy progression in mice was associated with the expression of the myomesin isoform normally found expressed at higher levels in the hearts of embryos, and this condition is also observed in humans [46]. Chronic pressure-overload cardiac hypertrophy is a common cause of dilated cardiomyopathy, and is associated with distinct alternative splicing due to altered expression of splicing factors [48].

### 3.2. Arrhythmogenic Right Ventricular Cardiomyopathy

The influence of alternative splicing on arrhythmogenic right ventricular cardiomyopathy is seen in the alternative splicing of genes such as the Neuroblastoma apoptosis-related RNA-binding protein (NAPOR) [49]. Alternative splicing of the plakophilin 2 gene, which links cadherins to intermediate filaments in the cytoskeleton in cardiac muscle, results in a seven nucleotide deletion in exon 12 that leads to a frame-shifted isoform with an extended open reading frame. The expression of this mutant isoform leads to ARVD [50]. Finally, dysregulation of RNA-binding proteins responsible for the regulation of alternative splicing are known to contribute to ARVD [51].

## 4. Alternative Splicing and Apoptotic Genes

A great number of apoptotic factors including “NF-κB” and *Bcl-2* genes undergo alternative splicing processes, which lead to the production of distinct protein isoforms with antagonistic functions, *i.e.*, pro-apoptotic and anti-apoptotic, respectively.

### 4.1. Serine-Arginine (SR)-Rich Proteins and Their Specific Kinases

The role played by splicing factors in the splicing of genes in the heart is also of interest in apoptosis related pathologies. As mentioned in the introduction Serine/arginine-rich (SR) proteins and the heterogeneous nuclear ribo-nucleoproteins (hnRNP) are two families of splicing factor proteins that are involved in the regulation of alternate splicing. Serine/arginine rich splicing factor/Splicing factor 2 ASF1/SF2 is a sequence specific splicing factor. It has been observed that this splicing factor is required for the normal development of the heart, and is involved in the splicing of troponin 2, Cypher and the Ca^2+^/calmodulin-dependent kinase IIδ (CaMKIIδ). The change in the expression of these isoforms is required for the well-coordinated switch from the immature embryonic functions of the developing heart into the fully functioning mature heart. For instance the absence of ASF/SF2 leads to the expression of the incorrect isoform of CaMKIIδ, resulting in [52].

The activity of splicing factors can be controlled by the state of their phosphorylation. Multiple kinases can phosphorylate the serine arginine residues of splicing factors and thereby influence their activity; these include Cdc2-like kinases, which can control splicing in this way during cell proliferation. In Section 2.3 the role of the Akt pathway in dilated cardiomyopathy (DCM) was discussed [19]. Akt2, a member of the Akt subfamily of serine/threonine kinases that contain SH2-like (Src homology 2-like) domains is able to use Cdc2-like kinases as a substrate and regulate their activity through phosphorylation [53]. The splicing activity of another splicing factor, Srp40, is also controlled through Akt activity [54]. The hnRNP A1 protein and ASF are both involved in the control of the alternative splicing of caspase-2 [55], which is discussed in Section 4.2.

### 4.2. Caspases

Caspases are the effectors of apoptosis and are present in the cell as inactive pro-caspases, which are activated in response to apoptotic stimuli. Three isoforms of pro-caspase-2 have been identified, these are the long pro-apoptotic isoform caspase-2L, the anti-apoptotic caspase-2L consisting of the pro-domain of caspase-2L and the short anti-apoptotic isoform caspase-2S [56]. Pro-apoptotic stimuli, including cyclohexamide, etoposide, pacritaxel and staurosporine, promote exon-9 inclusion upon splicing of the caspase-2 mRNA following the treatment of U937 cells, thus, increasing the ratio of caspase-2S/caspase-2L in a time-dependent manner [11]. Increased levels of the anti-apoptotic caspases isoform were identified in macrophage-derived foam cells of human atherosclerotic plaques and may represent a survival strategy for cells undergoing DNA damage [57]. The expression of these alternate forms of caspase-2 is controlled through the activity of the splicing factors SC35, ASF and hnRNP A1. The serine arginine rich splicing factors SC35 and ASF promote exon skipping, leading to an increase in the expression of the pro-apoptotic caspase-2L. The heterogeneous nuclear ribonucleoprotein A1 leads to exon inclusion, resulting in an increase in the expression of the anti-apoptotic caspases-2S [55].

Caspase-2 is known to play a role in apoptosis involved in cardiomyopathy. Studies using rabbits subjected to ischemia and reperfusion showed signs of apoptotic myocyte cell death with an increase in the proteolytic activation of caspases-2, -3 and -7 [58]. Furthermore, reperfusion rapidly induces a rapid increase in the activation of caspase-2 and an increase in caspase-2 cytochrome c dependent release [59]. Rapid right ventricular pacing enforces ventricular tachycardia. When this is used to induce heart failure in dogs the results are similar to the cardiomyopathic state observed in idiopathic dilated cardiomyopathy in humans. Following ventricular pacing in dogs, an increase in apoptosis was observed in the ventricular and atrial myocytes, both caspase-2 and caspase-3 levels were elevated following ventricular pacing [60].

### 4.3. The Bcl-2 Family

The general characteristics of the alternative splice variants expressed from apoptotic *Bcl-2* family genes including *Bax*, *Bcl-2*, *Bcl-g*, *Bcl-rambo*, *Bcl-x*, *Bfl-1*, *Bid*, *Bim*, *Mcl-1* and *PUMA* are well summarized [9]. The expression of many of these proteins is regulated by development, tissue distributions and subcellular localization. The functions of some *Bcl-2* family members having downstream mitochondrial consequences are regulated by protein phosphorylation [61]. The *Bcl-2* family is well studied and is divided into three subclasses depending on functional and structural features as reviewed in [62], namely, (i) anti-apoptotic subfamily proteins (Bcl-2, Bcl-x_L_, Bcl-w, and Mcl-1); (ii) pro-apoptotic members (Bax, Bak and Bok); (iii) pro-apoptotic Bcl-2 homology 3 containing (BH3)-only proteins (Bid, Bim, Bik, Bad, Bmf, Hrk, Diva, Noxa and PUMA; and (iv) newly identified BCL-2 family proteins (Bcl-2l10 (Boo/Diva), Bcl-2l12, Bcl-2l13 (Bcl-Rambo), Bcl-2l14 (Bcl-g), and Map-1).

**Figure 4:**
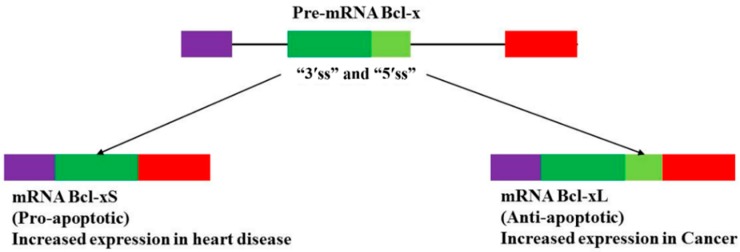
Alternative splicing of the *Bcl-X* gene transcript. Normal alternative splicing of the pre-mRNA *Bcl-x* gene yields a balanced ratio of mRNA pro-apoptotic Bcl-xS to anti-apoptotic Bcl-xL splice variants [66]. Abnormal alternative splicing of pre-mRNA Bcl-x may lead to overexpression of mRNA Bcl-xL splice variant in cancer [67] or overexpression of mRNA Bcl-xS splice variant in heart failure [68].

Alternative splicing of Bcl-x results in two isoforms, the pro-apoptotic Bcl-xS, short protein and the anti-apoptotic Bcl-xL, long protein (Figure 4). Staurosporine, a cytotoxic protein kinase C inhibitor increased the ratio of Bcl-xS/Bcl-xL leading to higher levels of apoptosis. Alterations of Bcl-x isoforms ratio by ceramide derivatives, hormones and growth factors have been noticed in various cell lines [63]. An increase in both Bcl-xL mRNA and its protein expression levels has been reported in patients with failing heart associated with cardiomyocyte apoptosis treated with a ventricular assist device [64]. Overexpression of the *Bcl-xL* gene by adenovirus vector resulted in an increase Bcl-xL protein expression and decreased doxorubicin (anti-cancer drug) induced apoptosis in neonatal rat cardiomyocytes [65]. Chemotherapeutic treatment of cancer with the anthracyclines daunorubicin and doxorubicin increases the risk of acute cardiac dysfunction and chronic cardiomyopathy with the end result being congestive heart failure.

The risks of cardiomyopathies developing, is further increased when Trastuzumab, a monoclonal antibody inactivating the transmembrane receptor tyrosine kinase HER2 or erbB2, is used in combination with daunorubicin and doxorubicin. Both of these treatments lead to the increase in the ratio of *Bcl-xS/Bcl-xL* and resulted in an increase in apoptosis [69]. Taken together, these findings reveal that alternative splicing of the apoptotic Bcl-x pre-mRNA may be altered during the induction of cardiomyocyte apoptosis in heart failure as well as during cardio-toxicity induced by some anti-cancer drugs. This results in the suppression of the transcription and expression of an anti-apoptotic Bcl-xL splice variant, whilst favoring that of a pro-apoptotic Bcl-xS isoform (Reviewed in [70]). Nevertheless, how alternative splicing of the *Bcl-x* gene is altered during cardiovascular disease is still not well understood and requires further investigations to validate functions of the abnormal splice variants and their impacts on the apoptotic pathways.

### 4.4. p53 and p53-Upregulated Modulator of Apoptosis (PUMA)

**Figure 5:**
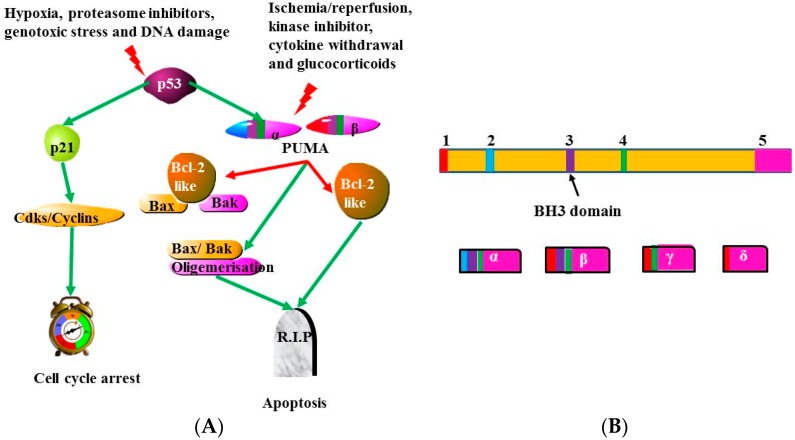
Illustrative diagrams of PUMA (BBC3) isoforms and activity. (**A**) PUMA is an essential inducer of p53-dependent and independent apoptosis. The tumor suppressor p53 can lead to cell cycle arrest or apoptosis depending on subcellular context and cell type. PUMA is needed for p53- dependent apoptosis mediated by hypoxia, DNA damage, proteasome inhibitors and genotoxic stress. Apoptosis is also induced by PUMA via p53-independent mechanism mediated by ischemia/reperfusion, kinase inhibitors, glucocorticoids and cytokine withdrawal (Reviewed in [71]); and (**B**) Alternative splicing of PUMA pre-mRNA yields mRNA PUMA α, β, δ and γ splice variants [20]. PUMA-α and -β splice variants contain BH3 domain, while PUMA-γ and δ have no BH3 domain. Thus BH3 domain in PUMA-α and -β variants plays an important role in the induction of apoptosis [20] and this may reveal that abnormalities in splicing of PUMA transcripts may result in either an increase or decrease in apoptosis due to exclusion or inclusion of BH3 domain in its splice variants.

The major tumor suppressor p53 is activated by various stimuli such as DNA damage, hypoxia and genotoxic stress. The activation of p53 leads to an increase in the expression of many genes regulating different cellular mechanisms including apoptosis, cell cycle arrest, senescence and autophagy. One of the p53-dependent apoptotic genes is a member of the BH3-only proteins of the BH2 family. This gene was named the *p53-upregulated modulator of apoptosis* (*PUMA*). PUMA activates apoptosis, whilst suppressing the function of anti-apoptotic genes. *PUMA* induced apoptosis via p53-independent mechanisms is initiated by ischemia/reperfusion, kinase inhibitors, glucocorticoids and cytokine withdrawal (Figure 5A) (Reviewed in [12,71,72,73,74]).

Alternative splicing of pre-mRNA PUMA (also known as BBC3) yields four splice variants, PUMA-α, -β, -δ and -γ in normal and tumor cells following chemotherapeutic treatment [75] (Figure 5B). Both PUMA-α and -β contain the BH3 domain, whereas PUMA-γ and PUMA-δ have no BH3 domain. Since the BH3 domain serves to promote apoptosis, both PUMA-α and -β may play a vital role in the induction of apoptosis [20]. This gives a probable mechanism for the role the splicing of PUMA may play in the occurrence of diseases. The incorrect splicing of PUMA may result to an increase or a decrease in apoptosis, as a consequence of exclusion or inclusion of BH3 domain. For example, PUMA-α and -β directly mediate mitochondrial induced apoptosis through binding to the BH3 domain of BAX, thereby facilitating its translocation and mitochondrial outer-membrane permeabilization to release cytochrome c [76,77]. Similarly, under UV induced apoptosis conditions, PUMA can also facilitate Bax translocation by directly interacting with Bax or by competitive binding of Bcl-xL [78].

PUMA has been demonstrated to be involved during ionizing radiation (IR) induced apoptosis through cytokine withdrawal and deregulated c-Myc expression, respectively [79]. Taken together these studies indicate that PUMA is implicated in the pathogenesis of cardiovascular disease through its involvement in the induction of cardiomyocyte apoptosis during ischemia/reperfusion injury in mice, while its deletion had been found to improve cardiovascular function [80,81,82]. In a process known as hypoxic post-conditioning, the damage to cardiomyocytes undergoing reperfusion can be decreased by a series of brief interruptions in reperfusion. It was reported that this action was due to the attenuation of ROS generation and the control of intracellular and mitochondrial Ca^2+^ overload [83]. However, it was found recently that hypoxic post-conditioning can lead to a decrease in cardiomyocyte apoptosis through the down-regulation of PUMA expression in a p53 related manner [84]. *PUMA* deletion was also found to attenuate apoptosis in cardiomyocytes in murine heart failure models [85], while ER induced stress led to an increase in PUMA dependent apoptosis in cardiomyocytes [86]. A recent study also demonstrated that mechanical stress brought on by hemodynamic overload increased the transcription of PUMA mRNA [87].

### 4.5. Bnip3 and Nix

Two members of the BH3-only subgroup of Bcl2-like proteins, Bcl-2/adenovirus E1B 19-kDa interacting protein 3 (Bnip3) and N-terminal interacting protein (Nix), have been found to play a critical role in the induction of apoptosis following cardiac hypoxia and hypertrophy. Both genes are expressed in the heart and are localized in the mitochondria [88]. Bcl-like proteins are also able to localize to the ER and in muscle tissue, the sarcoplasmic reticulum (ER/SR). Here the ER/SR store and release calcium, which can be captured by the mitochondria and used to stimulate energy production or initiate apoptotic and necrotic pathways [89].

Bnip3 is a member of the Bcl-2 family of proteins. Bnip3 has pro-apoptotic activity, which is regulated via hypoxia-inducible factor transcriptional complex and its mRNA expression is increased in response to a decrease in oxygen concentrations in cardiomyocytes and other cells [90,91].

Following hypoxia, ventricular myocytes show increased expression of Bnip3FL that leads to apoptosis and necrosis in ventricular myocytes [92]. Furthermore, short-term intermittent hypobaric hypoxia led to a decrease in the expression of Bnip3 and Bad proteins, and exerted protective effects on the hearts, whereas long-term intermittent hypobaric hypoxia increased the expression of Bnip3 and Bad proteins and led to increased wall thickness, abnormal myocardial architecture and apoptosis [93]. Bnip3 mediates permeabilization of mitochondria and release of cytochrome c via a unique mechanism that results in Bnip3 mediated mitochondrial matrix remodeling and swelling of the inner membrane [94]. This mitochondrial-based apoptosis is, however, dependent upon the shift of calcium from the ER to the mitochondria [95]. During hypoxic injury Bnip3 plays a role in the death of cardiac myocytes by acting on their mitochondrial function [96] and absence of activate Bnip3 suppresses mitochondrial related cell death of ventricular myocytes *in vitro* and *in vivo* [97]. The chemotherapy agent doxorubicin (DOX) was found to induce heart failure through a Bnip3 dependent mechanism involving mitochondrial disruption [98].

**Figure 6:**
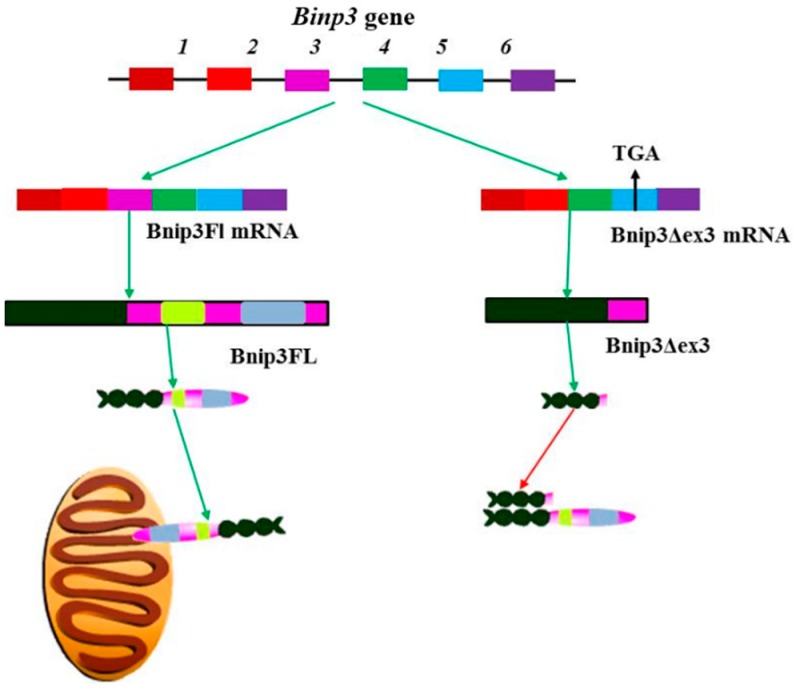
Alternative splicing of *Bnip3* and the function of the resulting isoforms. Bnip3 translation and expression is induced by hypoxia, with the alternate splicing of pre-mRNA to produce two isoforms, Bnip3FL and Bnip3Δex3. Bnip3Δex3 splice variant lacks both the BH3 and TM domains and is able to promote cell survival by heterodimerizing with BnipFL, preventing it from associating with the mitochondrial membrane. Generally, it is not fully understood how the alternate splicing of Bnip3 is controlled, but it is suspected that JNK signaling up-regulates apoptosis and BnipFL expression via the FOXO3a transcription factor.

Alternative splicing of the *Bnip3* gene is regulated by hypoxia in the ventricular myocytes, yielding two splice variants (Figure 6), the pro-apoptotic Bnip3FL containing both BH3 and transmembrane (TM) domains, and the anti-apoptotic Bnip3 splice variant Bnip3Δex3. This variant is generated through the alternate splicing of exon3 and lacks both BH3 and TM domains, (Figure 6) [92]. The expression of Bnip3Δex3 leads to diminished hypoxia induced apoptosis, and the consequent survival of ventricular myocytes [92]. The lack of the TM domain in Bnip3Δex3 implies that this isoform is unable to insert itself into the mitochondrial membrane. However, it is able to bind to Bnip3FL. This suggests a possible mode of action whereby, Bnip3Δex3 binds to Bnip3FL, preventing it from associating with the mitochondrial membrane, thereby, decreasing the ability of Bnip3FL to induce apoptosis [92]. Since hypoxia can induce a switch from apoptosis to cell survival via alternative splicing of the *Bnip3* gene, further studies are required to determine how the splicing of Bnip3 mRNA switches between these two isoforms during non-ischemic and ischemic cardiomyopathy [99,100]. It has been then suggested that Bnip3 expression may be mediated via JNK signaling. This is due to a JNK dependent increase in the activity of the transcription factor FOXO3a, which consequently increases the transcription of Bnip3. Therefore, JNK via Bnip3 mediates cardiomyocyte mitochondrial apoptosis and mitophagy, respectively [101].

Unlike Bnip3, which is induced by hypoxia, Nix expression is mediated by Gαq-induced hypertrophic stimuli [102]. G Protein Coupled Receptors (GPCRs) are involved in the signaling pathways controlling cardiovascular remodeling and alterations in Gα protein function is related to heart failure. This also explains the benefits of using antagonists of GPCRs such as β-adrenergic receptor blockers to treat heart failure [103]. Nix localizes to the mitochondria and sarcoplasmic reticulum, where it influences calcium release and regulates cell death. Nix mutants that could only be associated with the mitochondria or the ER, but not both, were equally efficient at inducing apoptosis. Therefore, Nix is able to induce apoptosis by altering mitochondrial membrane permeability. In this way, Nix acts as a linker of transcriptional and calcium-mediated signals for apoptosis [104].

**Figure 7:**
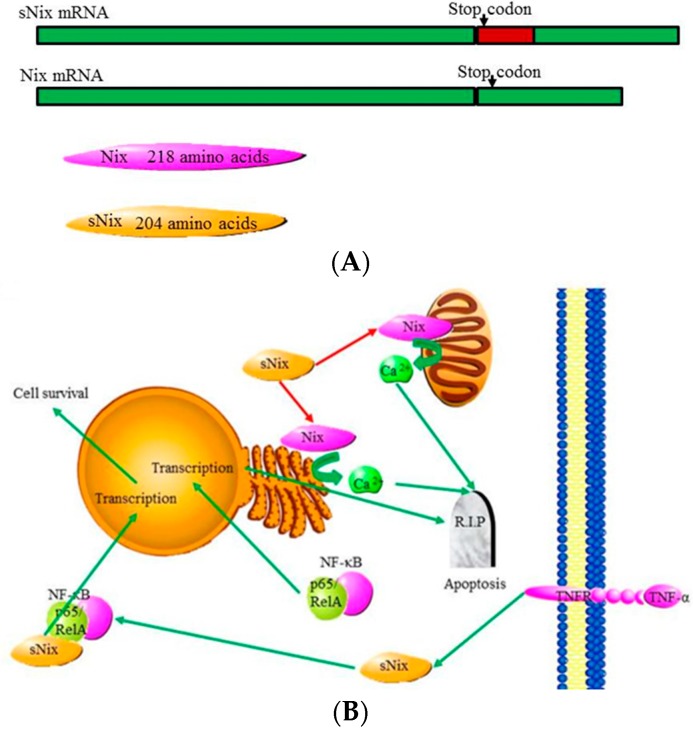
Alternative splicing of *Nix* and the function of the isoforms: (**A**) Alternative splicing of the *Nix* transcripts gives rise to a longer pre-mRNA with a retained intron. This retained intron contains a premature stop codon and results in a truncated protein product named sNix; and (**B**) Nix plays a pro-apoptotic role, while sNix plays an anti-apoptotic role by binding to and blocking Nix from interacting with the ER or mitochondrial membranes and inducing calcium release. sNix is also able to block apoptosis by binding to the “NF-κB” transcription factor and altering the genes targeted by the transcription factor [83].

Alternative splicing of *Nix* generates a variant named sNix. This short variant is able to diminish Nix induced cardiomyocyte apoptosis mediated by cardiac hypertrophy, a leader to heart failure (Figure 7A) [88,105]. Like most truncated Bcl-2 splice variants, sNix lacks essential targeting domains and can heterodimerize with the full length Nix and perform an inhibitory function [88]. However, sNix does not seem to be expressed at high enough levels to efficiently down-regulate Nix activity. Additionally, sNix localizes to the cytoplasm and nucleus, but not the mitochondria where Nix is localized. This suggests that sNix may down-regulate apoptosis in a unique fashion [105]. It was recently discovered that sNix protein only localizes to the nucleus once it is bound to the p65/RelA portion of the “NF-κB” transcription factor complex in response to TNFα. TNFα signaling normally induces the expression of pro-apoptotic genes via the “NF-κB” transcription factor complex. Once bound to the complex sNix negatively regulates the induction of pro-apoptotic gene expression, and reorients TNFα-induced “NF-κB” signaling to express cytoprotective genes (Figure 7B) [105].

## 5. Novel Therapeutic Strategies for Targeting Diseases Associated with Abnormal Splicing

Due to the various effects that the alternative splicing of apoptosis related genes or genes coding for the structural proteins that makes up the sarcomere, has on cardiovascular disease, various heart diseases can be mediated by errors in RNA splicing or its regulation. Therefore, targeting abnormalities or aberrant RNA processing is a valid target for drug development. For instance, digoxin, a purified cardiac glycoside, has a long history of use in the treatment of heart failure, but was only recently found to modulate alternate splicing in heart tissue. Other currently prescribed cardiotonics were also identified as affecting alternate splicing and demonstrate the effectiveness of drugs that affect splicing events [106].

**Figure 8:**
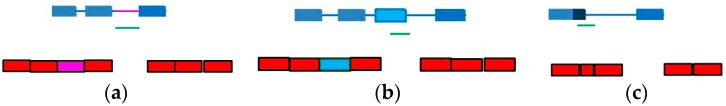
The use of splice-switching oligonucleotides (SSOs) to correct alternative splicing. SSOs are represented as green lines, the pre-mRNA sequences are in blue and the resulting transcripts are in red. (**a**) SSOs can be used to restore correct splicing of an aberrantly spliced transcript. Here an intron that has erroneously been included is removed through site directed splicing by the SSO leading to a transcript with the correct three exons; (**b**) SSOs can be used to produce a novel splice variant that is not normally expressed. Here they bind to and direct the splicing out of a previously included exon marked as light blue; and (**c**) SSOs can be used to manipulate alternative splicing from one splice variant to another. Here the SSO is being used to alter splice site recognition to exclude the portion of an exon, marked in black, using an alternative 5′ splice site. This mechanism can be used to down-regulate a deleterious transcript, while simultaneously up-regulating expression of a preferred transcript [3].

Implementation of alternative splicing knowledge as in the fields of phytochemical screening, pharmacogenomics, metabolic pathways and toxicity analyses are currently emerging [66,107]. Involving alternative splicing in pharmacogenomics studies can further lead to a decrease in the toxicity as well as improved efficacy and safety of drugs [108]. Notably, the dynamic nature, tissue and gene dependent variance of the splicing machinery and its interaction with pre-mRNA suggest a richness of possible targets for drugs that interfere with or redirect alternative splicing pathways for therapeutic purposes [2]. For example, splice-switching oligonucleotides (SSOs) (Figure 8) function to correct altered splicing by directing pre-mRNA splicing. This is achieved by binding sequence elements and blocking access of these transcripts to the spliceosome and other splicing factors. In this way, SSOs can restore correct splicing, produce a novel transcript or lead to the expression of proteins from an alternate transcript that may play a regulatory role [2]. Nanotechnology based medicine has the potential to minimize drug toxicity and improve drug delivery. This technology allows the utilization of antisense oligonucleotides for cancer therapy [109,110]. Nanotechnology has already been implemented in targeting some diseases including cancer (Reviewed in [111,112,113]). The applications, advantages and disadvantages of nanotechnology are reviewed in [114].

Although targeting alternative splicing of apoptotic genes may offer effective therapeutic strategies for the treatment of cancer and cardiovascular diseases. It is clear that challenges remain in the patients who might be suffering from both cancer (suppressed pro-apoptotic gene splice variants) and cardiovascular disease (overexpressed apoptotic gene splice variants). It is already known that many chemotherapeutic treatments increase the chances of cardiovascular diseases due to increased apoptosis [115,116,117]. Other novel therapeutic strategies that are currently emerging for targeting diseases associated with aberrant splicing include, siRNA-based drugs to silence gene expression, use of compounds that affect phosphorylation of splicing factors or stabilize putative secondary structures, a trans-splicing approach to replace mutated exons with wild-type exons and high-throughput screens to identify compounds that influence splicing efficiencies of target pre-mRNAs (reviewed in [118]). In order to implement any of these therapeutic strategies, it is vital that further investigation of the alternative splicing of apoptotic-related genes in diseased and healthy hearts be carried out.

## 6. Conclusion

Alternative splicing has emerged as an important field of study in heart disease. This is because the elucidation of the regulatory splicing factors of an apoptotic-gene and its specific splice variants in non-diseased and diseased hearts may facilitate understanding of the genetic-related heart diseases as well as the development of new therapeutic targets. Previously, the study of alternative splicing events was limited due to complex analysis, high cost and the false positives being identified in older, less sensitive techniques. Recently, new tools and technologies have been developed that allow for the economic and user-friendly analysis of alternative splicing events. One such technology, the Gene Array Analyzer (GAA) tool was developed from the Exon Array Analyzer, and allows for the detection of new splicing isoforms with limited bio-informatics training. The GAA was used to profile alternative splicing events during embryonic heart development in mice and it was discovered that many of the same splicing events that take place during development also take place during pathological conditions [119]. The decrease in sequencing costs and the advent of next generation sequencing will enable the identification of many more splice variants with a greater degree of accuracy.

Interestingly, medicinal plants are also revealing themselves as a promising source of agents for treating cancer and other diseases (Reviewed in [120,121]) and the screening of plant derived metabolites and compounds is emerging worldwide as a common technique to identify new drugs to combat diseases. Thus coupling studies of the abnormalities in alternative splicing of apoptotic gene with screening of traditional medicinal plants, which are known to treat apoptosis-related diseases, would be an approach that might offer cheaper alternative treatment for cardiovascular diseases.

## Abbreviations

Apoptosis repressor with caspase recruitment domain (ARC); Arrhythmogenic right ventricular cardiomyopathy (ARVC); Bcl-2/adenovirus E1B 19-kDa interacting protein 3 (Bnip3); CUG-BP, Elav-like family (CELF); Dilated cardiomyopathy (DCM); Exonic splicing enhancers (ESEs); Exonic splicing silencers (ESSs); G Protein Coupled Receptors (GPCRs); Heterogeneous nuclear ribo-nucleoproteins (hnRNP); Hypertrophic cardiomyopathy (HCM); Hypoplastic left heart syndrome (HLHS); Intronic splicing enhancers (ISEs); Intronic splicing silencer (ISSs); Long QT syndrome (LQTS); Myosin heavy chain β (MYH7); N-terminal interacting protein (Nix); p53-upregulated modulator of apoptosis (PUMA); Reactive oxygen species (ROS); RNA-binding proteins (RBPs); RNA polymerase II (RNAP II); Sarco/endoplasmic reticulum Ca^2+^ ATPase-type Ca^2+^ pumps (SERCA); Serine/arginine-rich (SR); Serum response Factor (SRF); Splice-switching oligonucleotides (SSOs); T type calcium channels (I_Ca_T); X-linked inhibitor of apoptosis protein (XIAP).
